# Supplementary material for: External Validation of a Mammography-Derived AI-Based Risk Model in a U.S. Breast Cancer Screening Cohort of White and Black Women
Source: Cancers (Basel). 2022 Sep 30;14(19):4803. doi: 10.3390/cancers14194803 (PMC9564051; doi:10.3390/cancers14194803)
Supplement: Supplementary file 1 [file cancers-14-04803-s001.zip › Supplementary Figures_rev1.pdf]

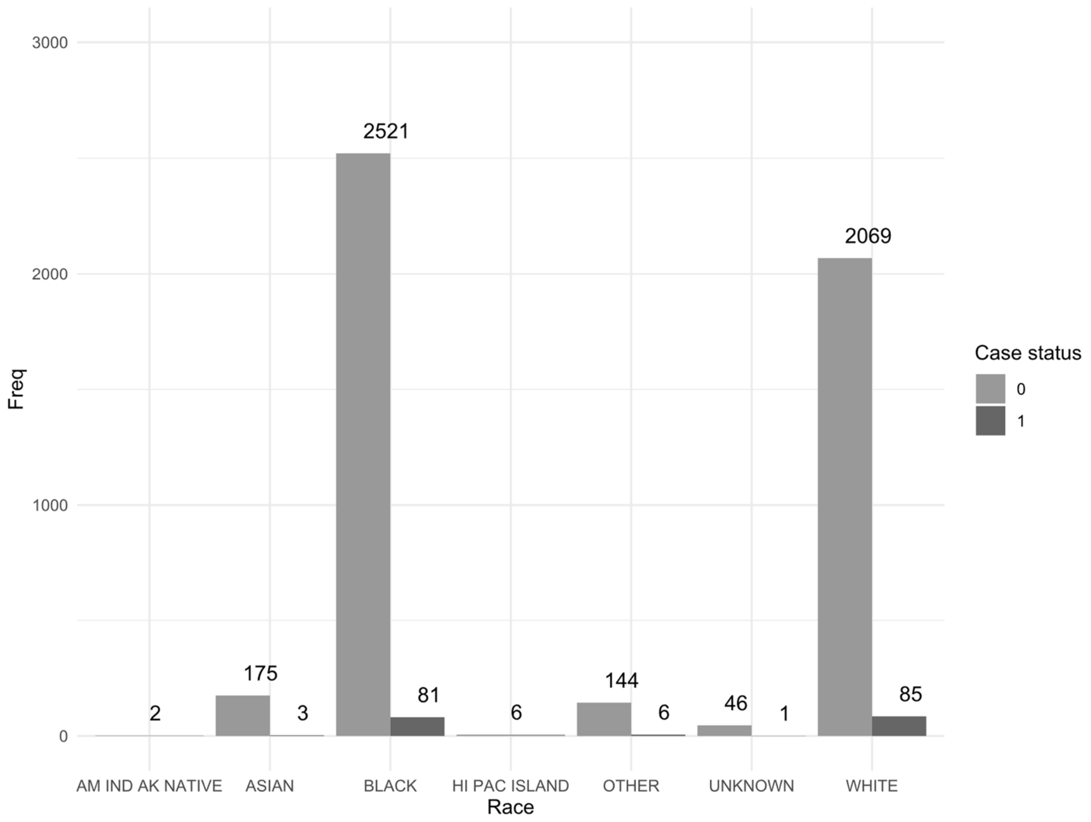

Supplementary Figure S1. Racial distribution of breast cancer cases and controls in study dataset.

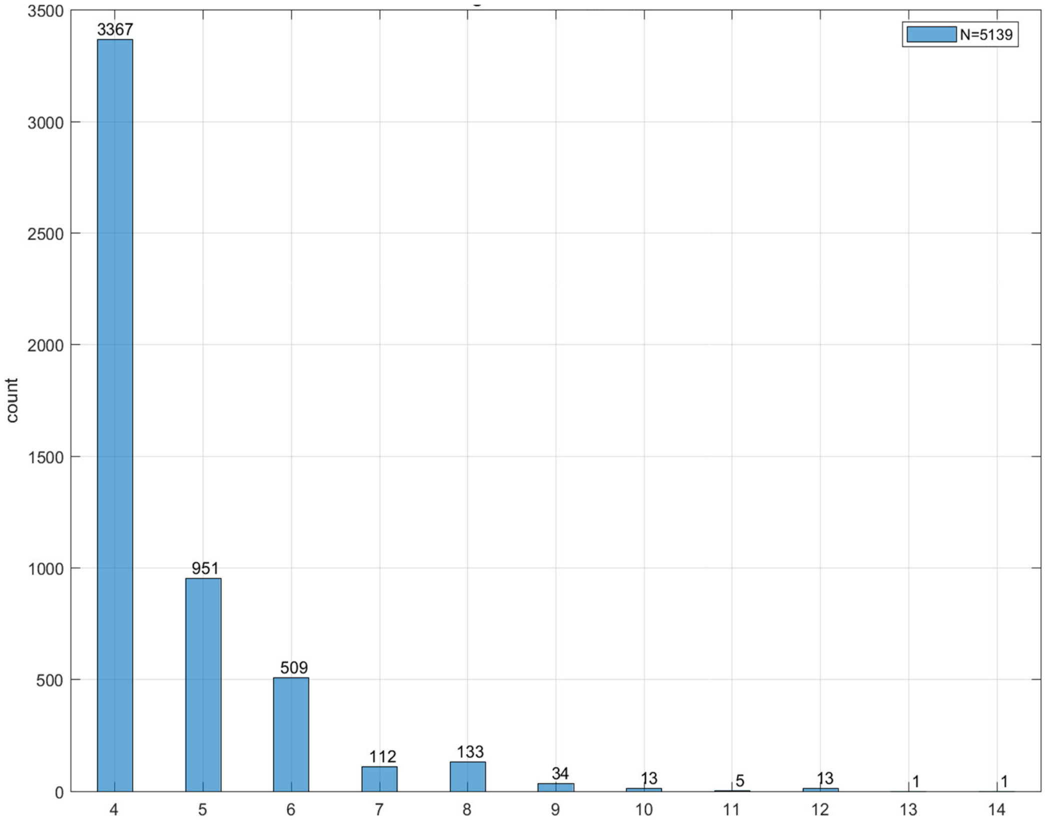

Supplementary Figure S2. Histogram of number of FFDM images per mammographic exam.

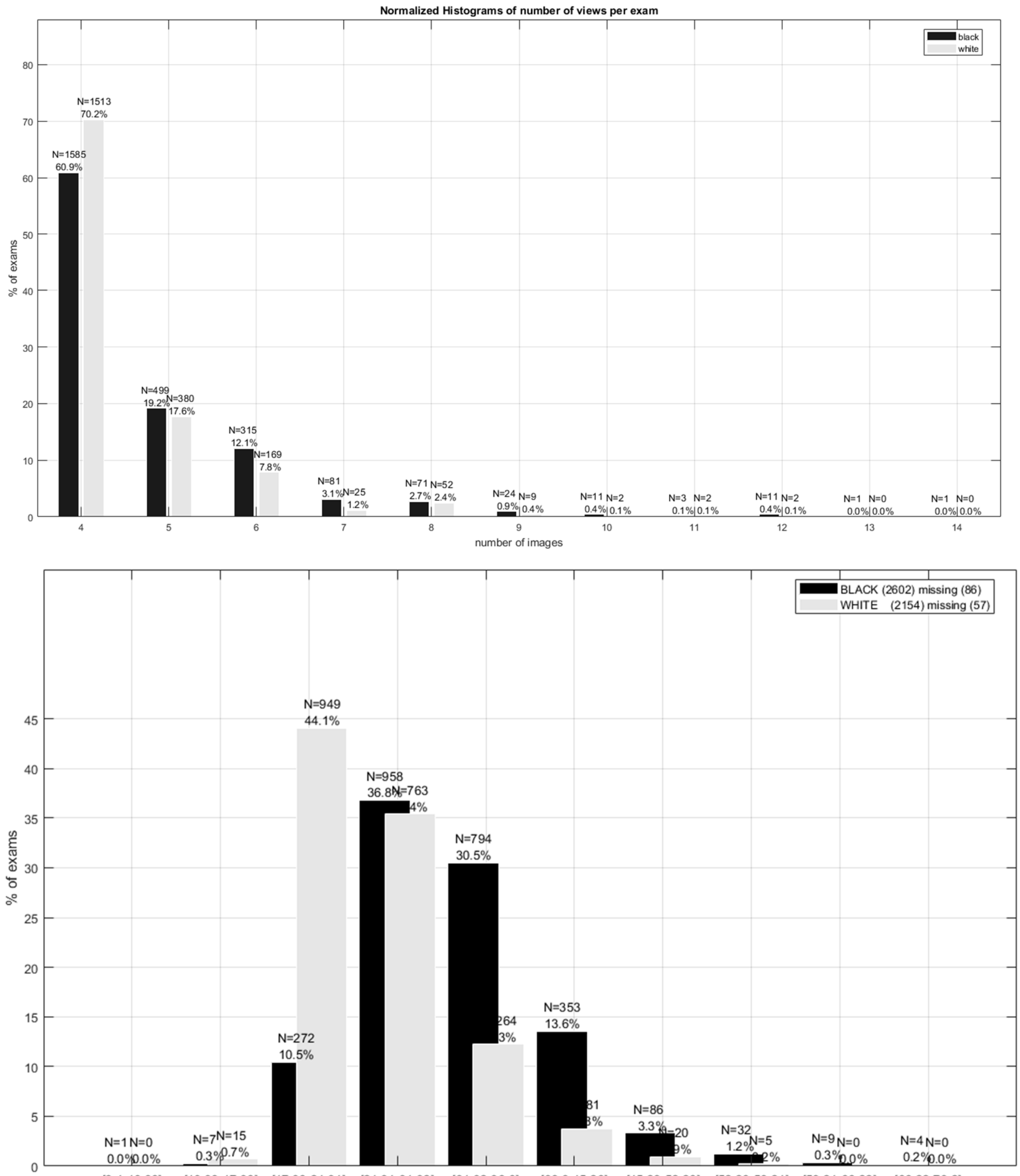

Supplementary Figure S3. Histograms of (top row) number of FFDM images per mammographic exam and (bottom row) BMI, for White and Black women.
