# Supplementary material for: External Validation of a Mammography-Derived AI-Based Risk Model in a U.S. Breast Cancer Screening Cohort of White and Black Women
Source: Cancers (Basel). 2022 Sep 30;14(19):4803. doi: 10.3390/cancers14194803 (PMC9564051; doi:10.3390/cancers14194803)
Supplement: Supplementary file 1 [file cancers-14-04803-s001.zip › Supplementary Tables_rev1.pdf]

Supplementary Table S1. Baseline characteristics in relation to racial subgroups.

Risk factor	Controls (N=4,963)			Cases (N=176)		
	White, N = 2,069[1]	Black, N = 2,521[1]	Other, N = 327[1]	White, N = 85[1]	Black, N = 81[1]	Other, N = 9[1]
Age at screening	57.09 (10.36)	56.29 (10.22)	54.46 (10.34)	59.58 (11.34)	61.13 (11.05)	63.72 (8.24)
BMI at screening	26.35 (6.06)	32.51 (7.47)	25.03 (4.81)	26.53 (6.02)	32.72 (6.30)	24.91 (3.48)
Missing BMI	53	81	28	4	5	1
Age >50 (postmenopausal)	1,500 / 2,069 (72%)	1,734 / 2,521 (69%)	198 / 327 (61%)	65 / 85 (76%)	65 / 81 (80%)	9 / 9 (100%)
Age at first child						
Nulliparous	595 / 1,886 (32%)	430 / 2,097 (21%)	65 / 277 (23%)	17 / 72 (24%)	13 / 61 (21%)	2 / 8 (25%)
< 20	73 / 1,886 (3.9%)	729 / 2,097 (35%)	20 / 277 (7.2%)	2 / 72 (2.8%)	21 / 61 (34%)	1 / 8 (12%)
20-24	287 / 1,886 (15%)	519 / 2,097 (25%)	42 / 277 (15%)	14 / 72 (19%)	12 / 61 (20%)	3 / 8 (38%)
25-29	477 / 1,886 (25%)	252 / 2,097 (12%)	76 / 277 (27%)	17 / 72 (24%)	9 / 61 (15%)	1 / 8 (12%)
>= 30	454 / 1,886 (24%)	167 / 2,097 (8.0%)	74 / 277 (27%)	22 / 72 (31%)	6 / 61 (9.8%)	1 / 8 (12%)
Missing	183	424	50	13	20	1
Family history of breast cancer						
No family history	1,609 / 2,048 (79%)	2,075 / 2,484 (84%)	265 / 322 (82%)	50 / 82 (61%)	58 / 75 (77%)	6 / 9 (67%)
One 1st degree relative	392 / 2,048 (19%)	379 / 2,484 (15%)	53 / 322 (16%)	24 / 82 (29%)	12 / 75 (16%)	3 / 9 (33%)
≥2 1st degree relatives	47 / 2,048 (2.3%)	30 / 2,484 (1.2%)	4 / 322 (1.2%)	8 / 82 (9.8%)	5 / 75 (6.7%)	0 / 9 (0%)
Missing	21	37	5	3	6	0
Number of prior biopsies						
0	195 / 610 (32%)	221 / 549 (40%)	18 / 56 (32%)	2 / 27 (7.4%)	2 / 16 (12%)	0 / 3 (0%)
1	269 / 610 (44%)	242 / 549 (44%)	26 / 56 (46%)	12 / 27 (44%)	9 / 16 (56%)	3 / 3 (100%)
2 or more	146 / 610 (24%)	86 / 549 (16%)	12 / 56 (21%)	13 / 27 (48%)	5 / 16 (31%)	0 / 3 (0%)
Missing	1,459	1,972	271	58	65	6
Atypical hyperplasia	25 / 210 (12%)	3 / 119 (2.5%)	2 / 18 (11%)	3 / 11 (27%)	0 / 5 (0%)	0 / 1 (0%)
Missing	1,859	2,402	309	74	76	8
BI-RADS density						
1	148 / 2,069 (7.2%)	455 / 2,521 (18%)	15 / 327 (4.6%)	4 / 85 (4.7%)	8 / 81 (9.9%)	1 / 9 (11%)
2	1,128 / 2,069 (55%)	1,520 / 2,521 (60%)	144 / 327 (44%)	39 / 85 (46%)	40 / 81 (49%)	4 / 9 (44%)
3	733 / 2,069 (35%)	519 / 2,521 (21%)	156 / 327 (48%)	41 / 85 (48%)	32 / 81 (40%)	4 / 9 (44%)
4	60 / 2,069 (2.9%)	27 / 2,521 (1.1%)	12 / 327 (3.7%)	1 / 85 (1.2%)	1 / 81 (1.2%)	0 / 9 (0%)

[1] Mean (SD); n / N (%);

N=46 controls and N=1 breast cancer case have missing information on race.

Supplementary Table S2. Detection and tumor characteristics at follow-up for all breast cancer cases, and in relation to racial subgroups.

Characteristic	All, N = 176[1]	White, N = 85[1]	Black, N = 81[1]	Other, N = 9[1]
Screening to diagnosis (days)[2],				
p1	107	115	107	152
p25	382	388	378	414
p50 (median)	423	440	409	486
p75	590	654	498	581
p99	762	751	763	678
Detection classification				
False negative-MR detected	7 / 173 (4.0%)	6 / 84 (7.1%)	0 / 79 (0%)	1 / 9 (11%)
False negative-PET detected	1 / 173 (0.6%)	1 / 84 (1.2%)	0 / 79 (0%)	0 / 9 (0%)
False negative-symptomatic	19 / 173 (11%)	9 / 84 (11%)	9 / 79 (11%)	1 / 9 (11%)
False negative cat3	2 / 173 (1.2%)	0 / 84 (0%)	2 / 79 (2.5%)	0 / 9 (0%)
True positive	144 / 173 (83%)	68 / 84 (81%)	68 / 79 (86%)	7 / 9 (78%)
Missing	3	1	2	0
Invasiveness				
In-situ	48 / 176 (27%)	26 / 85 (31%)	19 / 81 (23%)	3 / 9 (33%)
Invasive	128 / 176 (73%)	59 / 85 (69%)	62 / 81 (77%)	6 / 9 (67%)
Tumor size, invasive (mm)	15.91 (20.02)	11.67 (10.14)	20.93 (26.10)	5.86 (2.54)
<=10mm	108 / 176 (61%)	60 / 85 (71%)	39 / 81 (48%)	9 / 9 (100%)
>10 – 20 mm	43 / 176 (24%)	18 / 85 (21%)	24 / 81 (30%)	0 / 9 (0%)
>20 mm	25 / 176 (14%)	7 / 85 (8.2%)	18 / 81 (22%)	0 / 9 (0%)
In-situ grade				
Low-intermediate	35 / 49 (71%)	18 / 26 (69%)	15 / 20 (75%)	2 / 3 (67%)
High	14 / 49 (29%)	8 / 26 (31%)	5 / 20 (25%)	1 / 3 (33%)
Affected lymph nodes	31 / 135 (23%)	9 / 62 (15%)	22 / 66 (33%)	0 / 6 (0%)
Missing	41	23	15	3
Stage				
1a	88 / 175 (50%)	45 / 85 (53%)	36 / 80 (45%)	6 / 9 (67%)
1b	1 / 175 (0.6%)	1 / 85 (1.2%)	0 / 80 (0%)	0 / 9 (0%)
2a	19 / 175 (11%)	7 / 85 (8.2%)	12 / 80 (15%)	0 / 9 (0%)
2b	8 / 175 (4.6%)	0 / 85 (0%)	8 / 80 (10%)	0 / 9 (0%)
3a	3 / 175 (1.7%)	2 / 85 (2.4%)	1 / 80 (1.2%)	0 / 9 (0%)
3b	2 / 175 (1.1%)	1 / 85 (1.2%)	1 / 80 (1.2%)	0 / 9 (0%)
3c	2 / 175 (1.1%)	0 / 85 (0%)	2 / 80 (2.5%)	0 / 9 (0%)
4	4 / 175 (2.3%)	3 / 85 (3.5%)	1 / 80 (1.2%)	0 / 9 (0%)
DCIS	48 / 175 (27%)	26 / 85 (31%)	19 / 80 (24%)	3 / 9 (33%)
Missing	1	0	1	0
Advanced cancer	44 / 176 (25%)	13 / 85 (15%)	30 / 81 (37%)	0 / 9 (0%)
ER-positive	137 / 173 (79%)	70 / 83 (84%)	59 / 80 (74%)	7 / 9 (78%)
Missing	3	2	1	0
HER2-positive	20 / 146 (14%)	13 / 68 (19%)	6 / 69 (8.7%)	0 / 8 (0%)

Missing	30	17	12	1

[1] Mean (SD); n / N (%); N=1 woman has missing information on race.

Advanced cancers were defined as cancers of AJCC Pathological Prognostic Stage of 2A or greater.

[2] Percentiles of distribution of days from date of screening to date of diagnosis.

Supplementary Table S3. AI risk scores in study dataset: Distributions by case-control status in relation to racial subgroups.

Risk factor	Controls (N=4,963)			Cases (N=176)		
	White N = 2,069[1]	Black N = 2,521[1]	Other N = 327[1]	White N = 85[1]	Black N = 81[1]	Other N = 9[1]
Breast percent density[2]	30.15 (21.37)	21.58 (18.66)	31.96 (20.03)	33.96 (23.18)	30.53 (21.48)	20.94 (15.02)
Calcs malignancy	0.12 (0.14)	0.14 (0.17)	0.11 (0.14)	0.20 (0.20)	0.25 (0.23)	0.21 (0.26)
Masses malignancy	0.18 (0.19)	0.18 (0.20)	0.16 (0.18)	0.24 (0.24)	0.24 (0.24)	0.20 (0.23)
Calcs asymmetry	0.03 (0.04)	0.04 (0.06)	0.02 (0.04)	0.05 (0.07)	0.08 (0.09)	0.07 (0.11)
Masses asymmetry	0.05 (0.06)	0.05 (0.06)	0.04 (0.05)	0.08 (0.09)	0.07 (0.08)	0.05 (0.04)
AI 2-year risk, % (Q1,Q3)	0.79 (0.50,1.33)	0.80 (0.50,1.40)	0.62 (0.42,1.06)	1.19 (0.77,2.51)	1.53 (0.83,3.65)	1.18 (0.63,1.78)
Gail 5-year risk, % (Q1,Q3)[3]	1.61 (1.16,2.12)	1.30 (0.99,1.62)	0.85 (0.56,1.41)	1.82 (1.39,2.96)	1.36 (1.08,1.72)	1.55 (0.91,2.13)

Mean (SD); n / N (%); Median (Q1,Q3).

[1] N=46 controls and N=1 breast cancer case have missing information on race.

[2] For one breast in a control exam the percent density was not obtained, and the unilateral density result was used for the risk analysis.

[3] Gail risk was available on 166 cases and 4,894 controls.

Supplementary Table S4. Discriminatory performance (AUC) of AI risk model at two acquisition time-points. (1st acquisition timepoint) using the images acquired first for each FFDM view of the mammographic exam and (last acquisition timepoint) using the images acquired last for each FFDM view of the mammographic exam, in all women with >4 FFDM views and in racial subgroups.

Risk model	All women (66/1,706)		White women (31/610)		Black women (34/983)		p-value[2]
	AUC	95% CI	AUC	95% CI	AUC	95% CI	
1st acquisition timepoint	0.69	0.62 - 0.75	0.65	0.55 – 0.75	0.71	0.62 – 0.79	0.37
Last acquisition timepoint	0.71	0.64 – 0.77	0.68	0.58 – 0.77	0.72	0.64 – 0.79	0.40
p-value[3]	0.71		0.78		0.83		

Only women with >4 FFDM views are included.

Unadjusted AUCs. Confidence intervals estimated using bootstrapping. Permutation test tested for difference between AUCs in White and Black women for each model (p-value[2]) and between 1st and last acquisition timepoint of the same mammographic exam (p-value[3]).
